# Hip impingement: beyond femoroacetabular

**DOI:** 10.1093/jhps/hnv049

**Published:** 2015-07-16

**Authors:** Nikolaos V. Bardakos

**Affiliations:** Private Practice, 18, Kalamakiou Avenue, Alimos 174 55, Athens, Greece

## Abstract

In the last 20 years, femoroacetabular impingement has been at the forefront of clinical practice as a cause of hip pain in young adults. As arthroscopic techniques for the hip continue to evolve, the possible presence of a new group of conditions creating mechanical conflict in and around the hip joint (ischiofemoral, subspine and iliopsoas impingement) has recently been elucidated whilst interest in already known ‘impingement’ syndromes (pelvic-trochanteric and pectineofoveal impingement) is now revived. This article attempts to increase awareness of these relatively uncommon clinical entities by describing their pathomorphology, contact mechanics, treatment and published results available to present. It is hoped that such knowledge will diversify therapeutic options for the clinician, thereby improving outcomes in a small but not negligible portion of patients with previously unexplained persistent symptoms.

## INTRODUCTION

Femoroacetabular impingement (FAI) is established as an intra-articular condition creating mechanical conflict between the acetabulum and the femoral head–neck junction, ultimately resulting in labral tears and acetabular cartilage damage. Numerous causes (genetic, physical activity, paediatric hip diseases, malunited femoral neck fractures, iatrogenic) of FAI have been proposed [1]. From an anthropological standpoint, however, FAI is regarded as an inevitable evolutionary corollary to bipedal stance [2]. Indeed, FAI has been discussed in the anthropology literature for more than a century and lesions such as ‘Poirier’s facet’ and the ‘fossa of Allen’, corresponding to the better known today cam lesion and herniation pit, respectively, are believed to be related to hip extension, such that occurs in walking or running, and have been identified with high frequency in the fossils of ancient Greeks [3]. Nevertheless, it was through the work of the Bernese group, led by Reinhold Ganz, that the orthopaedic community gained a solid insight into the pathophysiology of FAI and its potential for osteoarthritic degeneration, when left untreated [4].

Advancements in hip arthroscopy have transformed it to a reconstructive technique, making it an all too commonly used tool for the surgical management of hip disorders. Access to the peripheral compartment [5] and the use of suture anchors [6] were the first necessary technical steps surgeons needed to comprehensively manage all components of FAI, yielding equally good results to those of open surgery [7]. Today, armed with modern diagnostic tools (3-D CT, delayed gadolinium-enhanced MRI of cartilage, dynamic computer analysis), researchers are exploring novel-related fronts, like the concept of impingement-induced instability and the role of femoral/acetabular version on outcomes [7–8].

Growing experience with hip arthroscopy and improved understanding of FAI, including the ways its treatment can fail, has led to the recognition of new disorders of the non-arthritic hip. Their nomenclature also includes the term ‘impingement’, potentially creating some confusion but, in reality, they often co-exist with FAI. In parallel, there is a resurgence of interest in the so-called trochanteric-pelvic impingement (TPI) which was previously somewhat neglected in the literature. This review aims to present the aetiology, diagnosis, treatment and results of each of them, with an emphasis on arthroscopic management.

## ISCHIOFEMORAL IMPINGEMENT

### Definition and anatomy

Ischiofemoral impingement (IFI) refers to the painful entrapment of the quadratus femoris (QF) muscle between the lesser trochanter and the ischial tuberosity [9–12]. The QF muscle originates from the external border of the ischial tuberosity and inserts onto the upper part of the linea quadrata (the line extending vertically downward from the intertrochanteric crest) of the proximal femur [13]. As its name implies, the QF muscle has a rectangular shape. It is bordered by the obturator externus anteriorly, the sciatic nerve posteriorly, the inferior gemellus proximally and the adductor magnus distally [11]. It acts synergistically with the other short external rotators but also serves as a secondary adductor of the hip [11, 14].

### Historical background

IFI as a clinical entity was first described in 1977 by Johnson, who reported on three patients complaining of pain following total hip replacement (THR) (*n* = 2) or proximal femoral osteotomy (*n* = 1) [15]. The main reported symptom was groin pain radiating distally to the inner thigh and knee and was uniformly exacerbated clinically in a position of combined adduction, extension and external rotation. All patients were successfully treated with open excision of the lesser trochanter through a medial surgical approach [15].

The case report by Johnson emphasized the role of IFI as a potential pain generator in the context of previous or upcoming hip replacement surgery. More than 30 years later, it was the report by Patti *et al.* [16] on a patient diagnosed with IFI in her non-operated hip that revived interest in this condition. Subsequently, several clinical papers on IFI have been published [9–12, 17–28].

### Pathomorphology/pathomechanics

**Fig. 1.**
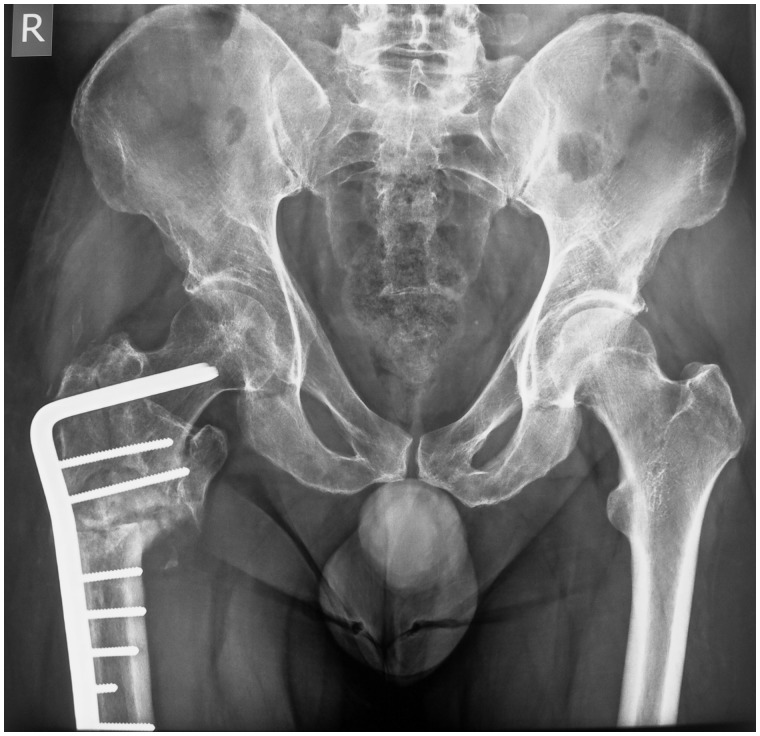
Anteroposterior pelvic radiograph following a valgus-derotational subtrochanteric osteotomy of the right hip for a malunited peritrochanteric fracture with involvement of the lesser trochanter (LT). The original injury and the valgus component of the corrective osteotomy have brought the right LT in close proximity to the ischium. Such a patient would be at risk for ischiofemoral impingement.

The salient pathomorphological feature of IFI is believed to be a reduced distance between the lesser trochanter and the ischium. Conditions originally proposed to reduce this distance included an intertrochanteric fracture with involvement of the lesser trochanter, a valgus proximal femoral osteotomy (Fig. 1) and hip arthritis with proximal or medial migration of the femoral head [15]. The overwhelmingly higher prevalence of IFI in women has led several investigators to consider the gender-related increased pelvic width of females as a fundamental predisposing factor [11, 16–18, 20, 24, 26] (Table I). Of particular value are the observations of the Bernese group, who used computer-assisted dynamic simulation to demonstrate that hips with Perthes disease or the combination of coxa valga and femoral anteversion (>25°) have a predilection for posterior extra-articular impingement in the form of IFI, more so than normal hips or those with FAI [29–30]. It should be stressed that two or more of the potential causes listed in Table I may need to co-exist for IFI to develop (e.g. an abductor muscle injury in a female patient with a medialized hip replacement).

**Table I.** Factors predisposing to ischiofemoral impingement

*Constitutional*
Increased width of female pelvis [11, 16–18, 20, 24, 26]
*Post-traumatic*
Peritrochanteric fractures with involvement of lesser trochanter [15]
*Post-operative*
Valgus-producing proximal femoral osteotomy [15]
THR with reduced femoral offset or medialized socket [15]
*Developmental*
Coxa profunda/protrusio acetabuli [15]
Coxa valga (± femoral anteversion) [29]
*Idiopathic*
Legg–Calvé–Perthes disease [30]
*Enthesopathy of hamstring origin* [22]
*Positional*
Abductor muscle injury causing uncompensated hip adduction during gait [20, 26]
*Expansile lesions*
Multiple hereditary or isolated exostoses [21]
*Senescence*
Age-related muscle atrophy [11, 21]

The author prefers the term ‘constitutional’ to ‘congenital’, as the latter, which has been used in previous literature, connotes a diseased state (THR, total hip replacement).

### Clinical findings

**Fig. 2.**
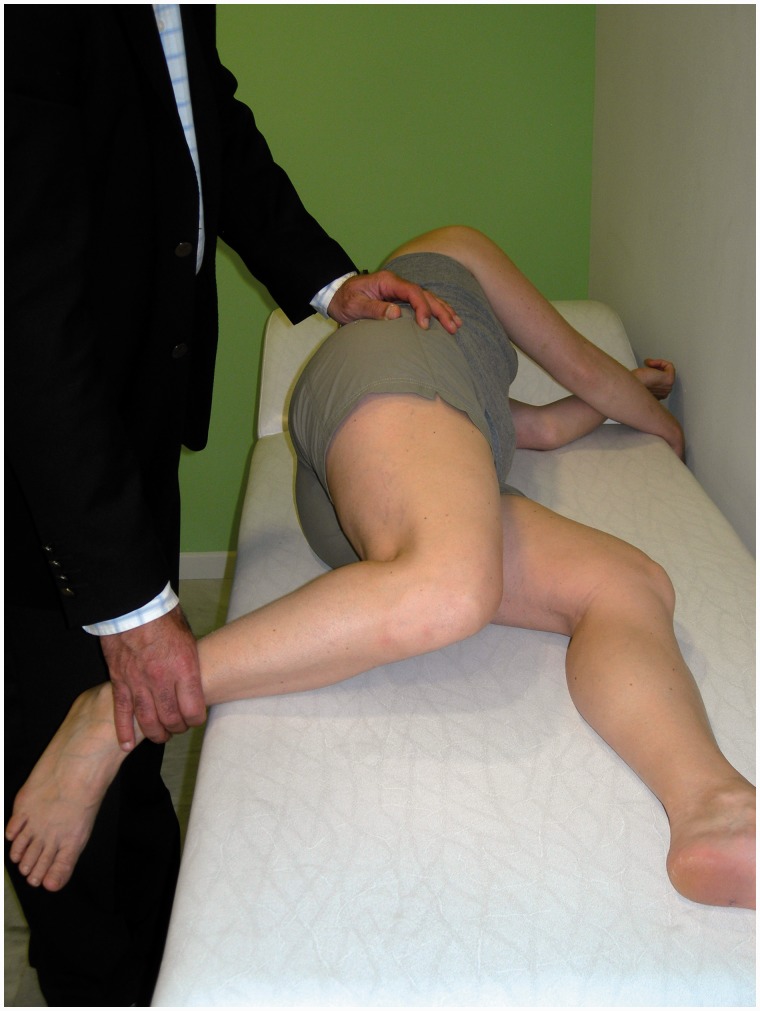
Clinical photograph demonstrating how combined hip extension/adduction/external rotation is performed during examination for ischiofemoral impingement. The unaffected extremity is brought forward, providing enough clearance for the hip to be adducted.

The clinical presentation of IFI is variable. Patients of any age may be affected: the youngest patients diagnosed with IFI were 11 years old [22–23]. The typical patient complains of pain of months’ or years’ of duration and a detailed history will often unveil a previous acute inciting traumatic episode or surgery [17]. The location of pain has been described in the groin and radiating distally to the medial thigh [15–16] but, in the setting of coxa valga, posterior buttock pain may be more common [29]. There is no pathognomonic clinical test for IFI; passive combined extension/adduction/external rotation (Fig. 2), a manoeuvre that approximates the lesser trochanter to the ischium, would be expected and has been reported to aggravate pain [12, 15] but differing painful positions, such as flexion/abduction/external rotation, have also been described [21]. Mechanical symptoms may be present, most notably snapping; indeed, it is now suggested that IFI should be added to the list of causes of a snapping hip [20, 31]. Finally, resisted external rotation may be painful [12] and, in cases with enlargement of the lesser trochanter, the extremity may be held in abduction, creating a functional leg length discrepancy [23].

### Imaging

Plain radiographs should be scrutinized for any visible cause, as outlined in Table I, that might alter the spatial relationship between the lesser trochanter and the ischium, bringing the two closer together. This finding is not always suggestive of impingement, however, because the proximal femur normally lies anterior to the ischium, anyway [23]. Heterogeneous sclerotic or subcortical cystic, geode-like, changes on the lesser trochanter or the ischium may be seen, indicating chronicity [11–12, 16, 21, 23]. Sonographic examination is not useful diagnostically [19], although therapeutic injections to the QF muscle have been performed under ultrasound guidance, which helps to avoid damage to the sciatic nerve [28]. MRI will confirm diagnosis if both (a) oedema of the QF muscle without disruption of muscle fibres and (b) a reduced distance between the lesser trochanter and the ischium, are seen. Oedema, seen as increased signal on an otherwise normal appearance on *T*_2_-weighted sequences [31], within the muscle belly itself with sparing of the myotendinous junction and involvement of the adjacent fat distinguishes IFI from muscle strains [17]. Additional findings may include oedema or partial tears of the hamstrings and the iliopsoas tendons and the formation of bursa-like formations [18]. Similar to rotator cuff pathology of the shoulder, fatty infiltration may ensue in long-standing cases [9, 11, 18]. It is worth remembering that MRI changes may take time to manifest; therefore, in a continuously symptomatic patient with an initially normal scan, it is perfectly reasonable to obtain a repeat scan after a few months [20].

In the first report on IFI, Johnson claimed, rather empirically, that the lesser trochanter and the ischium lie 2 cm apart [15]. Later, investigators have attempted to quantify the reduction of space in IFI by measuring the ischiofemoral (defined as the smallest distance between the ischial tuberosity and lesser trochanter) [18] and QF (defined as the distance from the superolateral surface of hamstrings to the posteromedial surface of the iliopsoas or lesser trochanter) [18] spaces and to provide threshold values for each. In three MRI studies, statistically significant differences between patients and controls were found for both spaces (mean ischiofemoral space, 13–15 versus 21–23 mm; mean QF space, 6.6–9 mm versus 12–15 mm). Cut-off values, determined by receiver operating characteristic curves, were estimated at ≤ 15–18 mm and ≤ 8–10 mm for the ischiofemoral and QF spaces, respectively [9, 18, 27]. In a study of 16 elderly (mean age, 83.6 years) cadavers (29 hips), the mean ischiofemoral space was 23.5 mm but the mean QF space (20.4 mm) was considerably larger than in the aforementioned MRI studies [26]. Differences in the study populations and the measurement techniques could account for this discrepancy. Interestingly, in a series of eight patients with painful snapping attributed to IFI, the ischiofemoral and QF spaces were normal in all (J.W.T. Byrd, personal communication).

### Treatment

Conservative treatment, in the form of non-steroidal anti-inflammatory agents or gabapentin, physiotherapy and CT-guided local steroid injections, is the mainstay of management of IFI [16, 22, 24–25]. In a high-volume hip arthroscopy practice, only one of more than 20 patients diagnosed with IFI needed surgical intervention [12]. Importantly, no attempt should be made to correct any functional leg length difference, as this will move the lesser trochanter closer to the ischium [23]. The successful use of ultrasound-guided prolotherapy (injection of an irritant to eliminate the nerve fibres associated with neovessels) has been reported for two patients at short-term follow-up [28]. Treatment of IFI in the presence of THR with excision of the lesser trochanter [15] should be viewed with caution; depriving the prosthetic joint of the iliopsoas may lead to instability, especially if a posterior surgical approach has been used during the index surgery [32]. Rather, consideration should be given to exchanging some of the prosthetic components in order to increase femoral offset [23]. The surgical treatment of IFI in native joints has been anecdotally reported using various techniques (Table II). Should the lesser trochanter be excised, the deep branch of the medial femoral circumflex artery (MFCA) must be protected in order to avoid osteonecrosis of the femoral head. A pre-operative angiogram is recommended to this end, especially when the local anatomy is grossly distorted, as in the presence of exostoses [23]. The endoscopic management of IFI has been reported by Safran and Ryu [12]. The author of the present review is also aware of an as yet unpublished series of eight patients undergoing endoscopic resection of the QF through a subgluteal approach, with mixed results (J.W.T. Byrd, personal communication).

**Table II.** Published outcomes of surgical treatment of ischiofemoral impingement (n, number of patients; LT, lesser trochanter; iHOT, International Hip Outcome Tool, reported as pre- versus post-operative score)

Authors (year of publication)	Study design	*n*	Age	Gender	Operation	Follow-up	Outcome
Ali *et al.* [20]	Case report	1	17	Female	Open excision of LT with reattachment of iliopsoas	10 weeks	Asymptomatic
Viala *et al.* [21]	Case report	1	37	Female	Open excision of ischial exostosis	6 months	Improvement of pain, appearing only after walking long distances
Ganz *et al.* [23]	Case series	14	11–63	10/14 female	Open distal advancement of LT	3.5 years (2–12)	LT healed; full hip flexion strength; subluxation disappeared
Safran and Ryu [12]	Case report	1	19	Female	Arthroscopic excision of LT with detachment of iliopsoas	2 years	No pain after a long recovery; hip flexion strength 5-/5; persistence of voluntary snapping; iHOT: 32 versus 85
Jo & O'Donnell [102]	Case report	1	17	Female	Endoscopic excision of LT without detachment of iliopsoas	4 months	Symptomatic relief within one week post-operatively, maintained during follow-up

The outcome in the study by Ganz *et al.* [23] applies only to the 8 of 14 patients who had Perthes disease and also underwent femoral head osteochondroplasty and relative femoral neck lengthening.

### The future

IFI is an infrequent condition. In a tertiary centre specializing in hip surgery, only 14 patients were diagnosed with IFI between 1997 and 2010 [23]. Guidelines for this diagnosis are mostly derived from radiological studies suffering from an inability to corroborate their data by operative findings [9, 18]. The description of patients with bilateral MR findings and unilateral symptoms [18, 22] suggests that imaging alone may over-diagnose IFI. Such an example is the report of a woman diagnosed with IFI on the basis of compatible imaging, even though her reported symptoms (sensory changes of the L_5_ dermatome, positive straight leg raise test at 30°) were more suggestive of lumbar radiculopathy [24]. It is expected that future research with use of dynamic imaging will enrich our knowledge on the pathophysiology and optimal treatment of this condition [11, 17–18, 26–27].

## SUBSPINE IMPINGEMENT

### Definition and anatomy

This term is used to denote the collision that may occur between an enlarged or malorientated anterior inferior iliac spine (AIIS) and the distal anterior femoral neck in straight flexion of the hip. Involvement of soft-tissue structures, such as the direct head of the rectus femoris and the iliocapsularis muscles, or the anterior hip capsule, in the impingement process has also been postulated [33, 34]. Among the causes implicated in this condition (Table III) [29, 34–37], valgus and anteverted femora represent a unique variant whereby, as a result of a distinct motion pattern (decreased adduction and external rotation) found in these hips, impingement may occur between a normally shaped AIIS and the greater trochanter or the anteroinferior femoral neck [29]. It should be noted that this is a proposition derived from specialized software simulation analysis and is the only cause with no clinical confirmation as yet.

**Table III.** Aetiology of subspine impingement

Cause	Comments
Apophyseal/rectus avulsions [35, 50]	Usually athletic individuals aged 14–23 years.
Developmental [35, 41]	In association with acetabular retroversion or in athletic individuals due to frequent powerful contractions of the rectus femoris.
Pelvic osteotomies [35–36]	When overcorrected
Valgus and anteverted proximal femur [29]	Predicted experimentally only

The recognition of subspine impingement (SSI) has rekindled interest in the anatomy of the AIIS and the rectus origin. In a study of 50 CT scans of young (mean age, 29.9 years) asymptomatic patients, the dimensions and orientation of the AIIS were measured [36]. The tip of the AIIS was found at a mean straight distance of 21.8 and 18.6 mm from the acetabular rim in men and women, respectively. Length, height and width of the AIIS were all larger in men than women but differences between genders ceased to exist when measurements were normalized for patient height and body-mass index, with the exception of width (11.9 mm versus 9.7 mm, *P* < 0.001) [36].

In a cadaver study of 11 male hips, the indirect head of the rectus was invariably located at the 12:00 o’clock position along the acetabular margin [35]. The location of the direct head was more variable but always extended between 1:00 to 1:30 (lateral margin) and 2:00 to 2:30 o’clock (medial margin). A bare spot, devoid of tendon, measuring ∼5 × 15 mm, was consistently present at the anterior and inferomedial aspect of the AIIS. The footprint of the rectus origin on the AIIS was found to be relatively broad (mean, 22 × 16 mm) and the mean distance of the AIIS tip to the acetabular margin was measured at 19 mm [35]. Similar mean dimensions (26 × 13.4 mm) of the footprint were reported in another study of six cadaveric specimens (12 hips) [38]. In that report, the psoas tendon and femoral nerve at the level of the pelvic brim were located at 19.3 and 20.8 mm, respectively, medial to the AIIS while the lateral circumflex artery was >5 cm away from the inferior aspect of the AIIS [38]. The most recent cadaver study by Philippon *et al.* [39] confirms these findings.

### Historical background

Among earlier clinical reports [40–42], all involving the use of open surgical management through a Smith-Petersen approach, Pan *et al.* [40] were the first to theorize in 2008 that a prominent AIIS might impinge against the femoral head–neck junction. In 2011, Larson *et al.* [34] coined the term ‘subspine impingement’ and for the first time described arthroscopic management in a small group of three athletes.

### Pathomorphology/pathomechanics

In a study of patients with FAI but no other deformity, Hetsroni *et al.* [43] proposed a classification system of AIIS morphology. With use of a dynamic testing software programme, they also provided evidence of progressive limitation of motion with increasing AIIS type (Table IV). When present, the area of collision of the AIIS against the femur was found distally on the anteroinferior neck. This is in contradistinction to FAI, where the femoral head–neck junction is involved [44]. No differences among AIIS types were found with respect to age, α-angle, and femoral/acetabular anteversion. Collectively, more than 80% of patients were found to have Types II or III morphology of the AIIS [43]. The less frequent Type I AIIS was not noted to cause impingement in this simulated analysis, although Hapa *et al.* [35] have stated impingement could occur in these hips during excessive flexion (e.g. ballet dancing).

**Table IV.** Classification system of the morphology of anterior inferior iliac spine and associated findings, as proposed by Hetsroni *et al*. [44]

Type	Relation to acetabular rim	Location of impingement	Range of movement	
Flexion				IR at 90° flexion
I	Above	Rim against neck (AIIS never involved)	120°	21°
II	Level	Distal AIIS against rim or distal area of anteroinferior neck	107°	11°
III	Below	Distal AIIS against distal area of anteroinferior neck	93°	8°

Differences in range of movement (mean values are reported) were all statistically significant except for the difference of internal rotation (IR) at 90° of flexion between AIIS Types II and III.

**Fig. 3.**
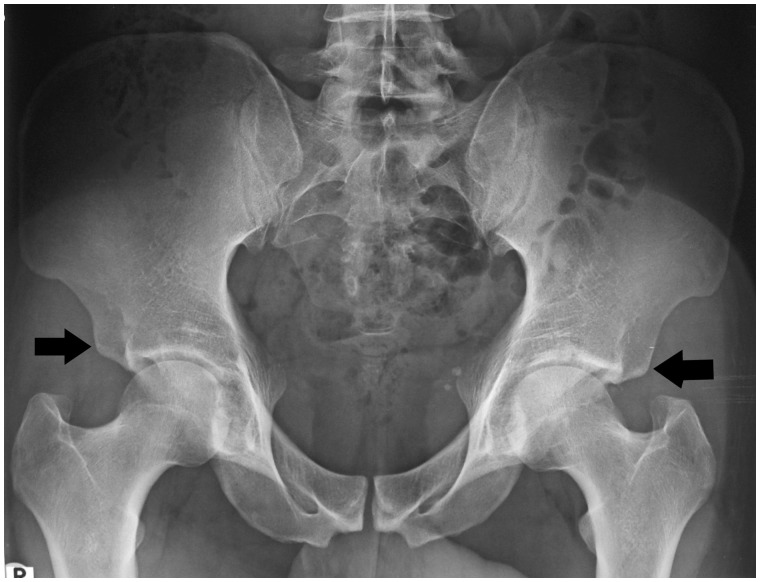
Anteroposterior plain radiograph of a 30-year-old male recreational football player. The AIISs (arrows) are at the level of (right hip) and below (left hip) the acetabular rim. Note the concurrent positive crossover signs and prominent ischial spines, suggesting apical retroversion of the acetabulae.

In another study of patients with FAI, however, Type I AIIS was by far the most common in both men (23 of 33) and women (23 of 25) [45]; it was also the only type observed in an anatomic study of 11 cadaveric male hips [35]. It appears that more research in large patient populations is needed to more precisely identify the true prevalence of the different AIIS types, although the evidence points to Type III being more common in men [43, 45] (Fig. 3).

### Clinical findings

The clinical presentation of SSI overlaps with that of FAI [40], in part because the two more often than not co-exist: in the largest (163 hips) series of patients treated arthroscopically for SSI, all were treated for FAI, as well [35]. Affected patients are usually young, active and involved in vigorous contact sports and may have a history of prior pelvic osteotomy or hip flexor injury [36, 40]. Unique signs and symptoms suggestive of SSI include a ‘grinding’ sensation of the hip, pain with kicking/sprinting activities, local tenderness on palpation of the AIIS, groin pain with straight flexion beyond 90° and only partial relief after an intra-articular test injection[34, 35, 46].

### Imaging

**Fig. 4.**
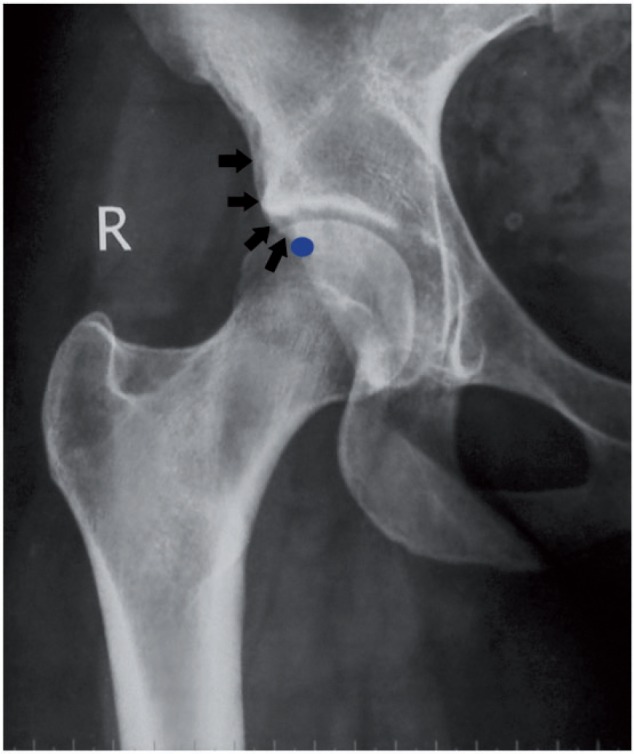
Close-up view of the right hip from an anteroposterior plain radiograph of a 19-year-old female with positive crossover sign. The point of crossover is marked by a blue dot. Arguably, the AIIS continues distally with round borders (black arrows) extending below the acetabular margin, potentially contributing to a false appearance of acetabular retroversion. Such a patient would require further investigation with computed tomography, to include 3-D imaging.

Suspected SSI is investigated with plain radiographs (anteroposterior of pelvis and false-profile of hip) and a CT scan [34, 35]. An MR arthrogram should also be obtained to rule out damage to the labrum and cartilage secondary to ongoing FAI. Calcific deposits within the origin of the rectus tendon, resembling calcific tendinopathy [47], and impingement cysts on the distal femoral neck may be noted, but the sine qua non of SSI is the finding of a distal and/or anterior extension of the AIIS [34]. A ‘spiky spur’ radiological appearance of the AIIS is seen in patients with previous avulsions, whereas smooth/round borders imply a developmental cause [46]. This is when radiographs should be interpreted very carefully, since SSI may present with subtle findings. Although the so-called ‘AIIS sign’ (AIIS outline clearly visible on a profile view) has been identified as one of four pelvimetric parameters associated with retroversion [48], a key study by Zaltz *et al.* [45] concluded that only 19 of 38 (50%) hips with a positive crossover sign had focal or true acetabular retroversion demonstrated on CT scans. In those hips with an anteverted acetabulum, a low-set AIIS was partially responsible for producing a false-positive crossover sign. In light of these findings, the differential diagnosis of SSI versus acetabular retroversion should never be based on radiographs alone (Fig. 4). Further evaluation with a CT scan, preferably with 3-D reconstruction, is essential in that regard, as it will clarify both the acetabular version and the anatomy of the AIIS [34].

### Treatment

Arthroscopic resection of an impinging AIIS, also termed ‘spinoplasty’ [49], has been reported by some experienced hip arthroscopists recently [34–36, 46, 49]. The diagnosis of SSI is confirmed arthroscopically by the presence of anterior focal synovitis and labral bruising in the area of the AIIS, as well as the presence of bony accumulation, representing the distal extension of the AIIS, on the acetabular rim [34, 35]. The surgical technique is predicated by the anatomy of the AIIS: the recognition of a consistent bare spot [35] on the AIIS confirms previous knowledge that the direct head of the rectus originates from the upper half of the AIIS only [41]. Using standard portals, the AIIS is exposed by capsular dissection from 1:30 to 2:00 o’clock. Care should be taken to refrain from dissecting too medially, or one risks direct injury to the femoral nerve and/or fluid extravasation into the intra- or retro-peritoneal space [38]. The bone is decompressed with a 5.5 mm burr. Although more may be needed in post-traumatic cases, bone removal of up to 1.5 cm in cephalad and anterior directions is usually adequate [35]. This amount of resection corresponds roughly to the size of the bare spot, minimizing the risk of rectus detachment. On the other hand, a window through the tendon fibres may be required for more extensive resection, yet without any adverse sequelae [35, 46]. Because of the extra-articular location of the AIIS, hip distraction can and should be used intermittently [49]. The post-operative administration of non-steroidal anti-inflammatory agents for prophylaxis against heterotopic ossification is recommended [46].

### The future

The published outcomes of arthroscopic treatment of SSI (Table V) are consistently good with no recurrences or complications and with documented preservation of the rectus femoris function as a hip flexor. However, they are hampered by the fact that SSI surgery is rarely performed in isolation, normally being one of many concomitant procedures, typically for FAI. To present, only one case of isolated arthroscopic AIIS decompression has been reported [34]. The same study reports on the single documented revision case after arthroscopic FAI surgery attributed to untreated SSI, with significant improvement post-revision [34]. Moreover, only results from high-volume hip arthroscopists are available, rendering the generalizability of results still unknown. Future research of higher level of evidence should investigate in more detail the interaction of SSI and FAI and provide guidelines as to when to address one or both at the same sitting.

**Table V.** Published outcomes of hip arthroscopy for subspine impingement (n, number of patients; HHS, Harris hip score; mHHS, modified Harris hip score; VAS, Visual Analogue Scale score; NAHS, Non-arthritic Hip Score; SF-12, Short Form-12 score)

Authors (year of publication)	Study design	Prevalence	*n*	Age (range)	Male gender	Sports involvement	Follow-up (months)	Outcome
Larson *et al.* [34]	Case series	N/A	3	23 (21–31)	33%	100%	16 (12–18)	HHS: 75.7 versus 93.7VAS: 6.2 versus 1.1
Matsuda and Calipusan [49]	Case report	N/A	1	13	Yes	Yes	18	NAHS: 22 versus 98
Hetsroni *et al.* [46]	Case series	0.7% (10/1370)	10	25 (15–44)	100%	100%	14.7 (6–26)	mHHS: 64 versus 98
Amar *et al.* [36]	Case report	N/A	1	20	Yes	Yes	6 weeks	Pain relief at 6 weeks
Hapa *et al.* [35]	Case series	Not reported	150 (163 hips)	28 (14–52)	50%	Not reported	11.1 (6–24)	mHHS: 63.1 versus 85.3; SF-12: 70.4 versus 81.3; VAS: 4.9 versus 1.9

Outcome is reported as mean pre- versus post-operative functional and pain scores, when available.

## ILIOPSOAS IMPINGEMENT

### Definition and anatomy

The iliopsoas is best known for causing what has been termed ‘internal snapping’ of the hip. This condition produces an audible, occasionally painful, clunk during movement of the hip from a flexed to an extended position, as the tendon’s movement from medial to lateral is interrupted by either the iliopectineal eminence or the femoral head [50]. Impingement of the iliopsoas against the prosthesis has been known as a complication of THR for almost 20 years [51] and impingement on the osteophytic acetabular rim was implicated as the cause of pain in a patient with degenerative hip arthritis [52]. In the modern sense, the term ‘iliopsoas impingement’ (IPI) is used to describe the mechanical conflict between the iliopsoas and the labrum, resulting in distinct labral lesions directly anteriorly (3:00 and 9:00 o’clock for right and left hips, respectively).

The psoas muscle originates from the 12th thoracic and all five lumbar vertebrae. The iliacus muscle originates from the iliac crest and the inner table of the ilium. The two merge to form the iliopsoas muscle, which has a musculotendinous insertion on the lesser trochanter, although some muscle fibres of the iliacus attach directly on the lesser trochanter and proximal femur [53–54]. In studies of fresh-frozen and embalmed cadavers, it has been shown that the iliopsoas is composed of ∼40% and 60% tendon and muscle, respectively, at the level of the labrum [53, 55]. At the insertion on the lesser trochanter these proportions are reversed, while at the level of transcapsular release of the iliopsoas through the peripheral compartment, the tendinous (53%) and muscular (47%) compositions of the iliopsoas are roughly equal [53]. A smaller muscle, termed the iliocapsularis, is consistently found adjacent and just lateral to the iliopsoas, originating from the inferior facet of the AIIS and the anteromedial hip joint capsule to insert 1.5 cm distal to the lesser trochanter [39, 56]. This may represent the ilioinfratrochanteric muscle identified in a previous cadaver study [54].

### Historical background

IPI on the anterior labrum was first reported in 2007 in patients undergoing revision hip arthroscopy as a cause of failure of the primary procedure [57]. Four years later, the same team published a retrospective study providing the first comprehensive report of this condition [58].

### Pathomorphology/pathomechanics

The intimate anatomical and functional relationship between the iliopsoas and the hip has been highlighted in previous studies. Using fresh-frozen cadavers, Alpert *et al.* [55] were able to show that the iliopsoas tendon directly overlies the anterior capsulolabral complex at the 2:00 to 3:00 o’clock position. This has been confirmed in a study examining findings of MR arthrograms [59]. In a passive kinetic experiment with use of 25 osseoligamentous cadaveric specimens, the investigators revealed the phasic heterogeneity that characterizes the function of the psoas major muscle: maximum pressures were consistently recorded in extension and upon the femoral head, which served as the pulley for the psoas in extension and early flexion; the psoas lost contact with the femoral head at a mean of 14° (range, 7°–19°) and, in turn, with its actual pulley, the highest point of the iliopectineal eminence, at 54° of flexion (range, 42°–67°) [60]. Similarly, peak tensile forces were found between 15° and 30° of flexion and decreased substantially thereafter. The authors of that study concluded the psoas major functioned more as a stabilizer of the femoral head and erector of the trunk at smaller (0°–45°) hip flexion angles whereas its function as a hip flexor was only evident at 45° to 60° [60].

Theoretical explanations for the focal anterior injury have been proposed and have included (i) a tight iliopsoas, causing impingement in extension, in accordance with the kinetic model described previously [60], (ii) a scarred iliopsoas, most commonly caused by chronic internal snapping, causing a repetitive traction injury to the labrum and (iii) a hyperactive iliocapsularis [58]. The latter explanation appears less credible, though, given the current evidence that the iliocapsularis becomes hypertrophic only in dysplastic hips [61].

### Clinical findings

Following the first clinical report on IPI [58], four more papers on this subject have been published [59, 62–64]. Overall, the five studies share common features with respect to patient demographics (young age, preponderance of females, involvement in sports). Although the clinical presentation of patients was not described in one [63], reported signs and symptoms have been non-specific and have included activity-related anterior hip pain with focal tenderness over the iliopsoas [58, 64] and discomfort in the sitting position[59, 64]. The impingement test has been uniformly positive [58, 59, 62, 64], other signs, such as the C-sign [62], scour sign [59, 64], flexion-abduction-external rotation (FABER) [59, 64] and pain with straight leg raising [59, 64] being encountered, too. A minority of patients with IPI may present with snapping. The finding of an anterior labral tear on MR imaging is helpful in resolving the diagnostic challenge in these.

### Imaging

These patients normally do not have any osseous pathomorphology on their plain radiographs [58, 62]. In an attempt to analyse the radiological features of IPI on MR arthrograms, Blankenbaker *et al.* [59] compared 23 patients (23 hips) with IPI documented at arthroscopy with 24 age- and sex-matched controls (24 hips) who underwent hip arthroscopy for other reasons. Although this study is limited by low levels of inter-observer agreement, labral tears from 2:00 to 3:30 o’clock were found more often in patients with IPI (reader 1: 20/23 versus 13/24, *P* = 0.024; reader 2: 18/23 versus 10/24, *P* = 0.017). For labral tears at the 3:00 o’clock position, substantial false-positive (reader 1: 54.2%, reader 2: 41.7%) and non-negligible false-negative rates (reader 1: 13.1%, reader 2: 21.7%) were reported. Moreover, some labral tears in the setting of other diagnoses were also seen to extend up to the 2:30 o’clock position. Of other radiological criteria investigated, only the lateral dip of the iliopsoas tendon at the level of the anterior labrum was found by one reader to be more common in the IPI group (*P* = 0.036). In the IPI group only, a trend was found for a smaller width of the tendon in women (10.5 mm versus 11.2 mm, *P* = 0.051) [59]. It should be stressed, however, that in the absence of established clinical or radiological criteria, the diagnosis of IPI can only be confirmed arthroscopically [64].

### Treatment

**Fig. 5.**
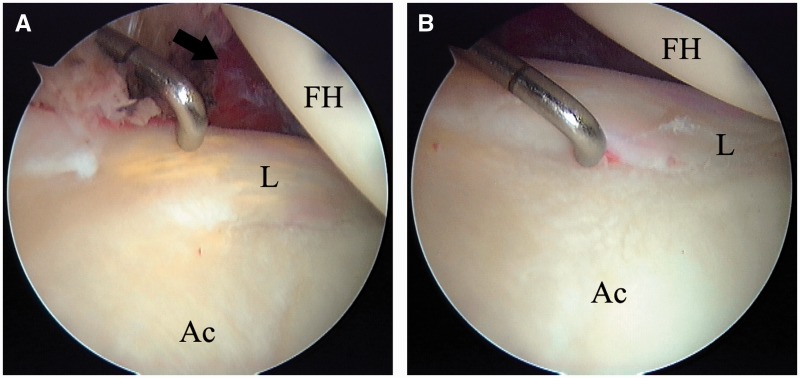
Arthroscopic image of a discoloured, degenerate anterior labrum with inflammation of the adjacent capsule (black arrow) (**A**). Probing of the chondrolabral junction reveals an early labral detachment (**B**). FH, femoral head; Ac, acetabulum; L, labrum.

IPI is effectively treated arthroscopically. The typical intra-operative finding is an isolated injury of the anterior labrum at the 3:00 o’clock (9:00 o’clock for left hips) position (Fig. 5) [58]. Management of the labrum (repair or debridement) is dictated by the pattern of injury: the labrum may be torn, degenerate or just bruised, flattened and inflamed. The presence of inflammation in the vicinity of the anterior labrum and the iliopsoas has been termed the ‘IPI sign’ [58]. It must be noted, however, that placement of suture anchors in this direct anterior position is technically more demanding than in anterosuperior locations typically found in FAI [63]. The iliopsoas, at times visible as it bulges through the adjacent capsule [63], is exposed through a transcapsular approach. It is freed of capsular adhesions and tenotomized with an arthroscopic biter, a beaver blade or radiofrequency probe. Simultaneously pulling the tendon into the joint and cutting it with an arthroscopic shaver has been suggested as a safety tip to maximize the distance of the tenotomy from the anterior neurovascular bundle [63]. Alternatively, tenotomy may be performed through the peripheral compartment of the joint [62]. Repair of the capsule is not required.

Results of arthroscopic treatment of IPI have been gratifying (Table VI). When care is taken to release only the tendinous portion of the iliopsoas, full flexion strength at 3 months is anticipated [62, 63]. Few complications have been reported, the most serious being one case of post-operative osteonecrosis of the femoral head [64]. Transient tendinopathy of the rectus has been described as common in one study [63]. This may call for gently exercising active flexion during early rehabilitation, until the iliopsoas heals.

**Table VI.** Published outcomes of hip arthroscopy for iliopsoas impingement (n, number of patients; HHS, Harris hip score; mHHS, modified Harris hip score; HOS, Hip Outcome Score; ADL, activities of daily living)

Authors (year of publication)	Study design	Prevalence	*n*	Age (range)	Female gender	Sports involvement	Follow-up (months)	Outcome
Domb *et al.* [58]	Retrospective (prospectively collected data)	5.6% (36/640)	25	25.1 (15–37)	92%	80%	21 (min. 12)	HHS: 61.6^†^ versus 87.2ADL HOS: 73.9^†^ versus 92.5Sport HOS: 51.6^†^ versus 78.8
Tey *et al.* [62]	Case report	N/A	1	37	No	Yes	3	Asymptomatic at final follow-up
Blankenbaker *et al.* [59]	Case-control	2.9% (23/800)	23 (study group), 24 (control group)	35 (16–57)	83%	N/R	12	mHHS: 43 versus 86; 3 recurrences
Cascio *et al.* [63]	Retrospective	3.7% (26/700)	26	19 (12–25)	95%	100%	Min. 6	HHS*: 70 versus 94; 1 recurrence
Nelson and Keene [64]	Retrospective (prospectively collected data)	10.7% (32/300)	30	35 (15–57)	80%	27%	24	mHHS: 43 versus 88; 3 recurrences

Outcome is reported as mean pre- versus post-operative functional and pain scores, when available. The studies by Blankenbaker *et al.* [59] and Nelson and Keene [64] come from the same research group and may overlap in their patient populations. ^†^Pre-operative scores available for eight patients only. *Pre- and post-operative scores available for 16 patients only.

### The future

A special subset of patients with IPI is comprised by these who present with concurrent snapping. Nelson and Keene [64] have been the only to describe detailed outcomes in those and have reported recurrent internal snapping in three of five such patients. Proposed causes of inferior outcomes or failure after arthroscopic iliopsoas tenotomy include increased femoral anteversion [65] and the presence of a bifid [66] or even a triple-banded tendon [67]. Nelson and Keene [64] attributed the recurrences to the cross-sectional anatomy of the iliopsoas at the level of the labrum, as described previously: because more of the musculotendinous unit remains intact after a labral-level, compared with a lesser trochanteric, tenotomy, tendon retraction is ∼5 mm less in the former; consequently, tendon regeneration is to be expected [68]. Although more research is needed, current evidence suggests that patients with IPI and snapping should be forewarned that snapping may recur after iliopsoas tenotomy through the central compartment. In these cases, a revision distal tenotomy, which has reliably shown very low recurrence rates for pure internal snapping [50], has been successfully utilized [64].

## TROCHANTERIC-PELVIC IMPINGEMENT

### Definition and anatomy

The greater trochanter (GT) and the femoral head and neck share a common growth plate, starting laterally as the greater trochanteric apophysis and continuing medially as the physeal plate of the proximal femur [69]. However, blood supply differs between the two, being extra-capsular for the GT and intra-capsular for the medial twothirds of the proximal femoral epiphysis [70]. Any insult to the intra-capsular perfusion, such that typically occurs in Perthes disease, affects the development of the femoral head and neck only. Trochanteric-pelvic impingement (TPI) suggests the painful abutment of a high-riding GT against the ilium during hip abduction in extension. In addition to Perthes disease, sepsis, slipped upper femoral epiphysis, osteonecrosis complicating trauma or treatment of hip dysplasia, adolescent osteonecrosis, previous varus intertrochanteric osteotomies and skeletal dysplasias may all produce similar deformities [71–74].

### Historical background

The first comprehensive description of TPI and its treatment is attributed to Jani, who noted an increase in the neck-shaft angle of the proximal femur following lateral advancement of the GT in 44 patients [71, 75]. The cornerstone of management of TPI in our era has been the introduction by Ganz *et al.* [76] and his team of the safe surgical dislocation of the hip.

### Pathomorphology/pathomechanics

**Fig. 6.**
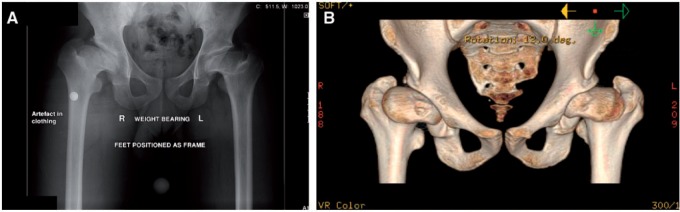
Anteroposterior plain radiograph (**A**) and corresponding 3-D CT (**B**) of a 20-year-old man showing the full spectrum of sequelae of Perthes disease (coxa breva, coxa plana and coxa magna, relative coxa vara due to a high-riding greater trochanter, steep acetabulum) in adulthood. Both hips were affected in this patient.

Left untreated, a hip affected by any of the previously described conditions may develop a complex deformity comprising any combination of a short femoral neck (*coxa breva*), a widened, flattened, mushroom- or saddle-shaped femoral head (*coxa plana* and *coxa magna*), a high-riding GT (*coxa vara*) and secondary acetabular dysplasia with limb shortening at skeletal maturity (Fig. 6) [72].

Relative overgrowth of the GT compromises the biomechanics of the hip joint in two ways. The hip functioning as the fulcrum of a lever system [77], there is diminished efficiency of the abductor mechanism, owing to the shortened resting length and lever arm of the gluteus medius and minimus muscles. In turn, this is known to increase the contact pressures across the joint [78, 79]. Furthermore, the GT and the ilium are brought closer together; during abduction, there may not be enough space to accommodate the GT, which then may abut against the outer wall of the iliac wing, causing painful impingement [71]. In severe deformities, the tip of the GT may be in contact with the posterior acetabular wall even in the resting position [80].

### Clinical findings

Patients with TPI typically complain of greater trochanteric pain and easy fatigue during walking or standing. In more severe cases, patients may walk with an abductor lurch [81] and the Trendelenburg test is acutely positive. In milder cases, a limp is observed only when patients are asked to walk quickly and it is only the delayed Trendelenburg test that is positive. It should be noted that time and patience are required on the examiner’s part when performing the Trendelenburg test in order to avoid false-positive and false-negative results [82]. The ‘gear-stick sign’ (passive abduction limited in extension but full in flexion, as the GT clears the ilium by moving posteriorly) has been described as a clinical sign to differentiate TPI from other causes of limited hip abduction [71].

### Imaging

The diagnosis of TPI is easily confirmed on plain radiographs. The relationship between the GT and the femoral head has been assessed qualitatively with the articulo-trochanteric distance [83] or can be quantitated using the centre-trochanter distance, which is the distance that the tip of the GT lies above or below the level of the centre of the head (normal range, 7 mm above–17 mm below) [84]. In the field of limb deformity correction, the mechanical or the anatomical medial proximal femoral angle (MPFA) (normal range, 80° to 89°; mean, 84°) is preferred [85]. In a logistic regression model, the anatomical MPFA was found to be the most important independent prognosticator (*P* = 0.02; odds ratio, 20.6 [95% CI, 3.4–34.8]) of progression of osteoarthritis in hips with radiological features of FAI. The authors of that study postulated that abductor dysfunction, denoted by a reduced MPFA, would account for such a finding [86]. Corroborating this theory, in a group of patients treated for post-Perthes FAI, the single patient ending up with a hip replacement was the only not to have open osteochondroplasty combined with trochanteric advancement [87].

### Treatment

Conservative treatment of TPI has not been reported. Presumably, the structural abnormality (overgrown GT) that underlies this condition cannot be resolved with other than surgical means. In the past, isolated transfer of the GT was used to improve abductor function and resolve impingement. This procedure is also referred to as ‘relative neck lengthening’ because only the superior part of the femoral neck is elongated [80]. Purely distal or lateral transfers have been described, although the latest trend is to combine both [72]. The surgical technique involves a lateral approach over the GT with detachment of the vastus lateralis origin. Under fluoroscopic control, the GT is osteotomized with use of a guide wire along the line of the lateral neck and transferred to the desired location [74]. The magnitude of distal transfer should be just enough to make the tip of the GT level with the centre of the femoral head. Temporary fixation with K-wires [87] or drill bits [72] is followed by definitive fixation with two or three fully threaded 3.5 or 4.5 mm cortical screws [72, 80]. Proposed tips to facilitate the procedure include the release of the tendon of the gluteus minimus (for mobilization of the fragment) [80], the preparation of the bone bed on the lateral femur with an osteotome or a burr (to enhance union and prevent undue lateral prominence) [71, 87] and the use of washers [72], which provide a buttress for maximal interfragmentary compression without risk of fracture. A subcutaneous technique for transferring the GT has also been described [69].

Studies on isolated transfer of the GT have shown it effectively relieves pain and improves function. Rates of elimination of the acute Trendelenburg test have varied between 61 and 91% at 4.3 to 8.8 years of follow-up [71, 74, 88, 89], although a delayed positive Trendelenburg test may persist in severe cases [71]. Improved ability to walk or stand for prolonged periods is probably the single most predictable benefit of this operation, as it has been observed even in patients with a persistently positive Trendelenburg test [71, 90, 91]. On the other hand, this procedure appears incapable of halting the degenerative process in those hips [89]. The GT predictably unites in its new position and reported complications (transient abduction contracture caused by an overzealous transfer, soft tissue irritation by screws) are minor [71, 74, 90]. Factors carrying a worse prognosis are a history of multiple previous operations, severe coxa vara, technical errors in re-positioning the GT, the pre-operative presence of degenerative changes and an underlying diagnosis of hip dysplasia, as opposed to Perthes disease [71, 74, 91].

**Fig. 7.**
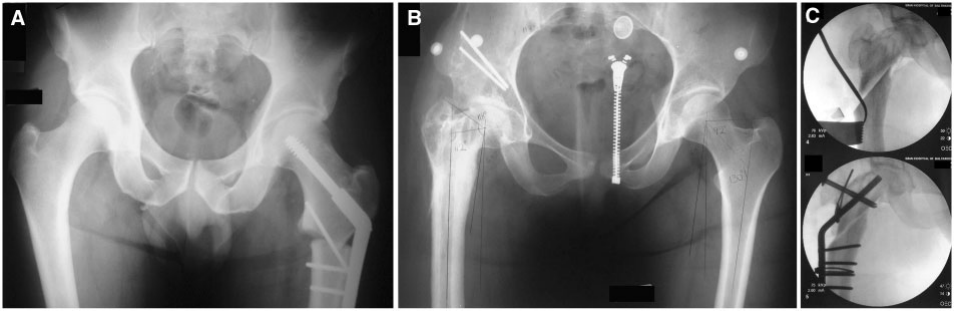
With a subtrochanteric valgus osteotomy (modified Wagner-type), the greater trochanter (GT) is essentially advanced distally and laterally. Note the intentional translation at the site of osteotomy (**A**). Anteroposterior pelvic radiograph of a 19-year-old woman with a history of multiple previous operations for a right congenitally short femur. The GT abuts the pelvis, despite a previous GT transfer. The neck-shaft angle measures 112° (**B**). The patient underwent a Morscher osteotomy (intra-operative radiographs shown) which involves sliding the femoral shaft distally and laterally along an osteotomy made at the desired neck-shaft angle. The GT is osteotomized and advanced at the same angle (**C**). Images provided by courtesy of Dror Paley, MD, FRCS(C).

Despite the value of isolated trochanteric advancement, modern management of patients suffering from the sequelae of Perthes or Perthes-like deformities usually involves an holistic approach. A detailed deformity analysis is mandatory pre-operatively: if the MPFA alone is abnormal, GT transfer will suffice but, in cases where both the MPFA and the neck-shaft angle are affected, a subtrochanteric valgus (modified Wagner-type) osteotomy is required. Alternatively, if the orientation of the femoral head in the acetabulum is to be maintained, a Morscher intertrochanteric osteotomy is preferable (Fig. 7) [69]. Co-existing acetabular dysplasia and/or retroversion may also need to be addressed with periacetabular or a Dega pelvic osteotomy [72, 73]. Tremendous progress in the operative treatment of this pathology has been made with the introduction of safe surgical dislocation of the hip by Ganz *et al.* [76], who later expanded our knowledge further by describing the development of an extended retinacular soft-tissue flap [73]. Previously unrealistic surgical goals, like the ability to re-shape a non-spherical femoral head, are feasible today [72, 73]. To further compound problems, such patients often suffer from intra-articular FAI [29, 92]; this may be treated equally well with open or arthroscopic techniques [87, 93]. Clearly, these cases are some of the most challenging in the field of joint-preserving surgery of the hip and should only be managed by surgeons who are both knowledgeable and skilful.

## PECTINEOFOVEAL IMPINGEMENT

### Definition and anatomy

This condition has received little attention in the literature. It has been theorized that, in select patients, symptomatic pectineofoveal impingement (PFI) may occur when an abnormally shaped medial synovial fold impinges against overlying soft tissue, primarily the zona orbicularis [94, 95].

**Fig. 8.**
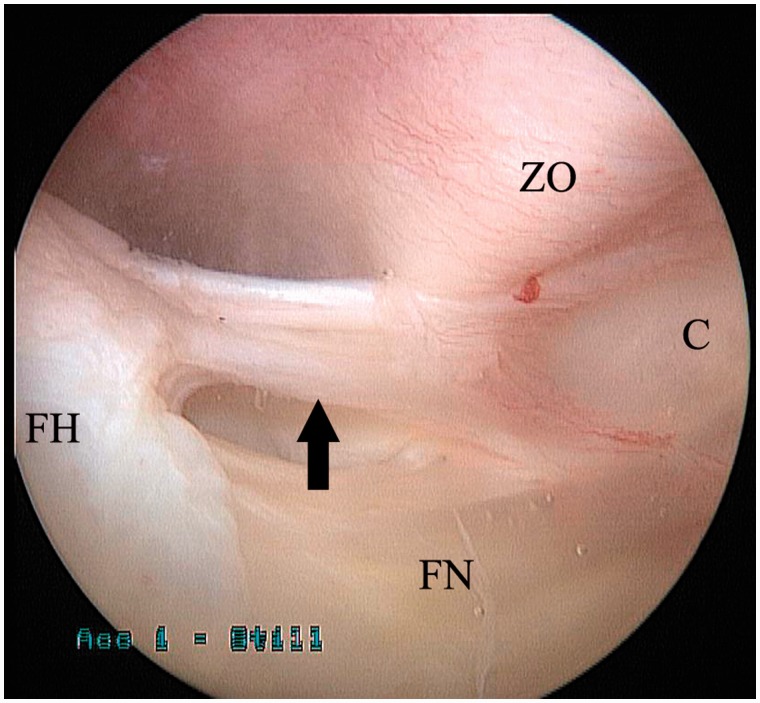
Arthroscopic image of the peripheral compartment of a hip joint showing the medial synovial fold (arrow) originating from the femoral head (FH) and inserting distally onto the capsule (C). The intimate anatomical relationship of the medial synovial fold with the zona orbicularis (ZO) is also demonstrated. FN, femoral neck.

The medial synovial, or pectineofoveal, fold represents a fibrous band located anteromedially on the femoral neck. It is consistently visualized during arthroscopy of the peripheral compartment of the hip, originating from the head–neck junction and inserting distally onto the capsule, crossing the zona orbicularis and iliopsoas tendon [94]. With rotational movements, it can be seen coming in close proximity to the zona (Fig. 8); with full flexion and external rotation, it may contact the labrum [94].

### Historical background

PFI was proposed in the previous decade by the French rheumatologists/arthroscopists Dorfman and Boyer, who developed this concept to explain the symptoms in a series of 10 patients (mean age, 26.8 years) undergoing diagnostic hip arthroscopy for inexplicable pain [95].

### Pathomorphology/pathomechanics

The hallmark of PFI is the presence of a thickened, fibrosed medial synovial fold. In this case, it may impinge against the overlying zona orbicularis and/or iliopsoas tendon [94, 95]. During flexion, it may impinge against the labrum. Arthroscopically, the typical appearance is that of localized synovitis, typically around the origin of the fold.

### Clinical and imaging findings

**Fig. 9.**
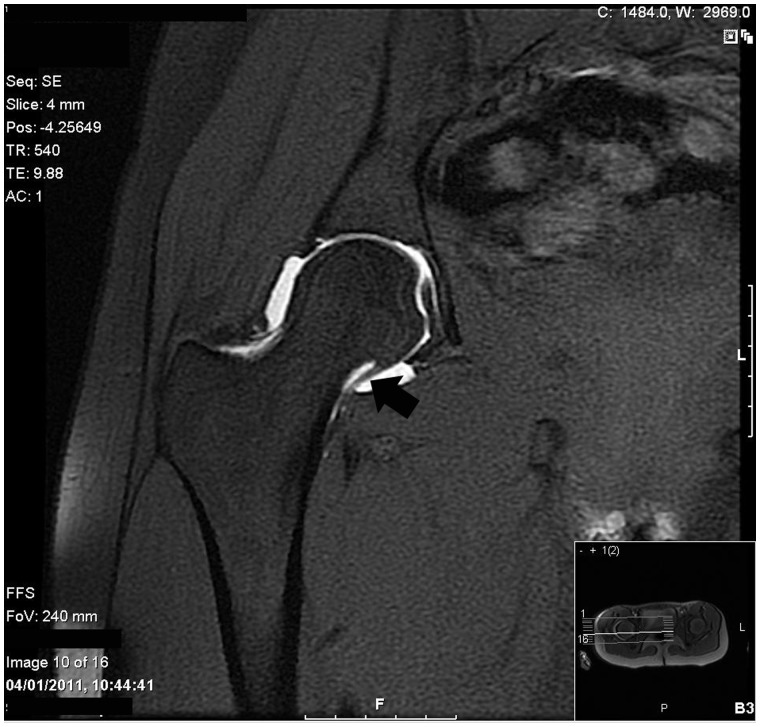
MR arthrogram (*T*_2_-weighted fat-suppressed sequence) of a right hip in the coronal plane, showing the medial synovial fold (arrow) adjacent to the anteromedial femoral neck.

PFI manifests clinically in a non-specific manner, with ill-defined hip pain aggravated by rotational movements and occasional feelings of hip blockage, but no snapping or clunking. The medial synovial fold is clearly visible on MR arthrogram as a band-filling defect (Fig. 9) to the extent that its dimensions can be accurately measured [96]. However, an MR diagnosis of PFI has not been reported to date. In effect, the diagnosis of PFI is arthroscopic.

### Treatment

This is accomplished by arthroscopic resection of the medial synovial fold, which may be carried out with a punch or a radiofrequency ablation device. Of the 10 patients treated by May *et al.* [95], five enjoyed a good/very good result; however, the method of assessing outcome was not reported. In the most recent report on these patients, the lasting successful outcome at >10 years’ follow-up was confirmed [94]. Symptoms remained unchanged in the remaining five patients. Patients with successful outcomes were typically engaged in sporting activities, operated on not too long (mean, 20 months) after symptom onset, in whom a thickened and fibrosed medial synovial fold with localized synovitis but no other pathology was found [95]. To the author’s knowledge, an elite female sprinter diagnosed with isolated PFI during hip arthroscopy has also enjoyed a fully successful outcome (J.W.T. Byrd, personal communication).

### The future

**Fig. 10.**
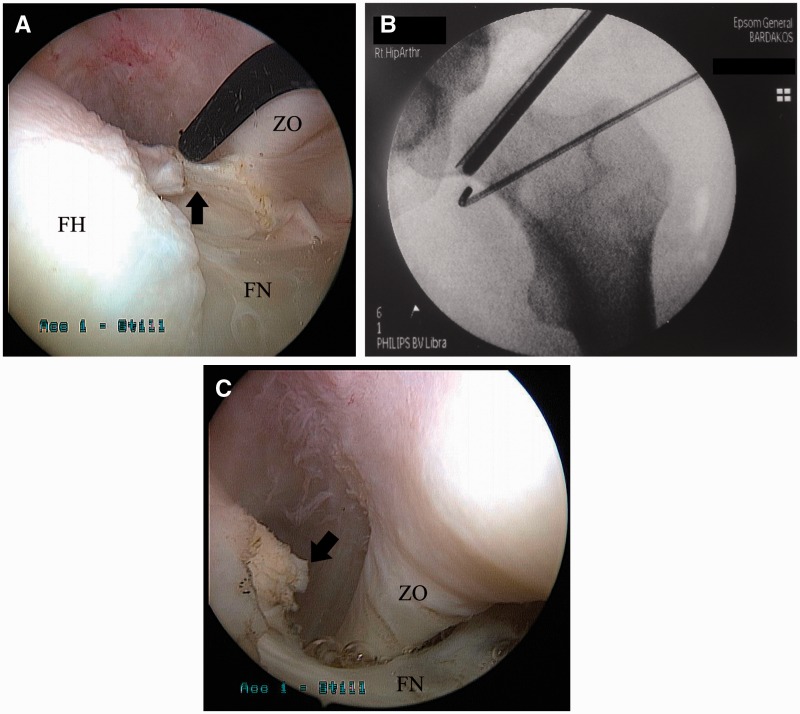
Arthroscopic technique of resection of the medial synovial fold. The patient is the one depicted in Fig. 8. The fold (arrow) is being resected with use of a deflectable radiofrequency probe (**A**). Corresponding fluoroscopic image showing the position of the probe against the medial femoral neck. External rotation of the hip (shown by the prominence of the lesser trochanter) facilitates access by bringing the fold more anteriorly (**B**). Final appearance with the medial synovial fold completely resected (arrow) (**C**). FH, femoral head; FN, femoral neck; ZO, zona orbicularis.

The precise nature, causes and even mere existence of PFI are viewed by most surgeons with scepticism and remain to be confirmed. To date, the medial synovial fold is best known for its utility as a landmark during arthroscopic transcapsular release of the iliopsoas through the peripheral compartment [97]. Nevertheless, hip arthroscopists should bear PFI in mind as a diagnosis of exclusion, in particular for those rare cases with focal pathology of and adjacent to the medial synovial fold in otherwise normal hips (Fig. 10).

## THE VALGUS HIP: A CAUTIONARY NOTE

Patients with coxa valga, defined as a neck-shaft angle > 135°, merit special emphasis. Excessive femoral anteversion is a commonly, although not universally, associated finding [98]. Coxa valga is seen in hip dysplasia and neuromuscular disorders or can present *de novo* as an isolated deformity [28].

A hip with a valgus and anteverted femur will typically demonstrate limitations in extension, adduction and external rotation [28]. It is the author’s of the current paper anecdotal experience that, not infrequently, rotation of the hip is neglected during clinical examination, especially by junior colleagues. This should never be the case. Although the supine position was proclaimed as advantageous for assessment of hip rotation in the classic article by Tönnis and Heinecke [98], others advocate the prone position to this end.

Although coxa vara has been implicated in the aetiology of classic FAI [99], coxa valga with antetorsion is considered to cause predominantly posterior FAI and dynamic anterior instability of the hip [28, 100]. As described throughout this manuscript, new insights in the pathomechanics of hips with coxa valga, with or without concomitant anteversion, have associated this deformity with ischiofemoral, subspine and ischiofemoral impingement. For each of these, distinct management strategies are available today; in the setting of coxa valga, however, their suitability should be considered carefully. For instance, recession of iliopsoas may lead to poor outcomes if it deprives the hip joint of an important dynamic stabilizer, thus decompensating an underlying occult anterior instability [33].

Assessment of the rotational profile of the lower extremities should always be part of a complete clinical examination of patients with hip or pelvic complaints. CT scans to objectively measure femoral and acetabular version are indispensable and today form part of the routine patient work-up in some tertiary referral centres [46]. Optimal decision-making for hips with coxa valga calls for consideration of the unique morphological and functional aberrations of this deformity. In a number of such patients, femoral corrective osteotomy may have to take precedence over other surgical options [28].

## CONCLUSION

A recent systematic review on most of the atypical impingement syndromes presented above concluded there was some evidence for improved outcomes following their surgical treatment [101]. With the exception of the long recognized TPI, our knowledge for the remaining entities remains limited, for reasons mostly relating to their low prevalence and their co-existence with typical FAI. However, their appreciation has undoubtedly opened new boundaries in the management of patients with non-arthritic hip pain. More research on these conditions is eagerly awaited. Being cognizant of these conditions may save delayed diagnoses and unnecessary operations in some of our patients.

## CONFLICT OF INTEREST STATEMENT

None declared.
